# The relationship between body mass index and children’s presentations to a tertiary pediatric emergency department

**DOI:** 10.1186/s13052-018-0476-y

**Published:** 2018-03-20

**Authors:** Valentina Ferro, Antonella Mosca, Francesca Crea, Maria Alessia Mesturino, Carla Olita, Andrea Vania, Antonino Reale, Valerio Nobili, Umberto Raucci

**Affiliations:** 1 Emergency Pediatric Department, Bambino Gesù Children’s Hospital, IRCCS, Piazza S. Onofrio 4, Rome, Italy; 2 Hepato-Metabolic Disease Unit, Bambino Gesù Children’s Hospital, IRCCS, Rome, Italy; 3 Centre for Pediatric Dietetics and Nutrition, Department of Pediatrics and Pediatric Neuropsychiatry Rome “Sapienza” University, Rome, Italy

**Keywords:** Obesity, Overweight, Childhood, Adolescence, Injury, Fracture, Emergency, Body mass index, Prevention

## Abstract

**Background:**

The child obesity and its complications are associated with an alarming increased health care use, including the emergency department (ED). We evaluated the effects of the obesity and overweight in children admitted to ED, especially in patients with injury diagnosis.

**Methods:**

A retrospective study of patients aged 6–18 years was conducted. Patients were categorized into normal weight (body mass index, BMI < 85th); overweight (BMI ≥ 85th e < 95th); obesity (BMI ≥ 95th). Multiple logistic analysis was used for estimation of risk factors associated with the BMI and to explore the association between injury diagnosis and BMI.

**Results:**

The predictive factors associated with obesity and overweight were school age (*p* <  0.001), male gender (*p* <  0.001) and number of visits for year (obesity: *p* <  0.001 and overweight: *p* <  0.05). Obese children were less at injury risk than normal weight (*p* <  0.05). In injury subset, fractures in school age were more likely to occur in obesity (*p* <  0.01). Dislocated fractures (*p* <  0.01) and fractures at lower extremity were more likely to occur in obesity and overweight (*p* <  0.05).

**Conclusions:**

School age children presenting to ED are more at risk of excess body weight than adolescents and are at higher fracture risk if obese and overweight. This has clear implication to support the efforts to reduce the obesity in childhood. The ED may represent a crucial setting for the early identification of these children and of co-morbidities related BMI ≥ 85th, and for a timely specialist referral of these children, especially if school age.

## Background

The worrying increase of obesity and overweight in children, resulting in what is now an epidemic, has drawn a raising attention to the potential short and long-term health consequences. In fact, this phenomenon places a significant burden on healthcare system as evidenced by increased hospital admissions for obesity-related diagnoses, lengthened hospital stay [1], higher medication use [2], and a greater frequency of outpatient clinic visits and emergency department (ED) visits as well [3], and a major use of the ED than primary care settings for routine medical care [4]. The hospital ED is frequently a major source of primary care and it might become a not less important setting for the screening, counseling and prevention of children at excess body weight risk. This is backed up by a previous study that shows parents and their children are interested in receiving education addressing healthier lifestyle choices and they feel that the ED is an appropriate setting for such an intervention program as well [5].

Further to obesity, another crucial challenge for health system is the unintentional injury that represents the leading cause of childhood death and disability involving a huge drain health and societal resources as well [6].

The relationship between obesity and injury is a relatively new and interesting field; most of the results are in the direction to draw a link between the overweight/obesity and the occurrence of injury even if some aspects remain unclear and contradictory [7]. The obesity and injury are two entities not to be considered separately [8], but they may interact with multiple implications on the child outcome and induce the need of enhancing the health surveillance and implement specific plans for child health safety. The aim of study is to describe the characteristics of children admitted to ED in relation to Body Mass Index (BMI) and to investigate the relationship between BMI and injury at ED. Finally, we would emphasize and promote the role of the ED as a strategic setting for screening and counseling, for the early identification of co-morbidities related BMI ≥ 85th, and for a timely specialist referral of these children.

## Methods

### Design study

A prospective observational study was conducted on patients aged 6–18 years admitted to ED at Children’s Hospital Bambino Gesù in Rome during period January 1th and October 31th, 2013. Exclusion criteria were: children aged under 6 years old and BMI < 5th percentile, children critically ill and parent of child participant did not consent the study.

The following data were recorded: sex, age, ethnic group, height, weight, waist circumference (WC), triage, symptom onset at admission, the number of visits in ED in the last year, outcome, length of hospital staying and diagnosis.

The age was categorized into two groups: school age (6–12 years) and adolescent (13–18 years).

The weight, height and WC measurements were performance by a health provider (pediatrician, medical resident, nurse) and not included cloths and shoes. The WC was calculated as the average of two measurements at umbilicus by a common meter.

We determined BMI of patients and the percentile, according to the WHO growth charts. Children were grouped into: normal weight (BMI ≥ 5th e < 85th percentile); overweight (BMI ≥ 85th e < 95th percentile); obesity (≥ 95th percentile).

The triage code to identify the severity of clinical condition at admission included: 1) yellow code (very urgent priority), 2) green and white code (urgent and non-urgent condition). The red code was excluded because corresponding to patients critically ill. The diagnoses were reported according to *International Classification of Disease, Ninth Revision codes*. Nevertheless, by the reason of great dimension of the sample we simplified for univariate and multivariate analysis into two diagnostic categories: a) injury, b) all others.

Then, we selected patients with injury diagnosis. We extracted also the following data. The type injury including: fracture; soft tissue injuries; laceration; strain, sprain, dislocation; concussion; multiple trauma/other mechanism. Body region injured: head/neck; face; upper extremity; lower extremity; trunk and multiple sites. The injury mechanism: fall/crash at leisure-sport activities; road traffic injury; fall between planes; static and passive mechanism (e.g. crush, electrocution; amputation etc.) and unknown mechanism. The study was approved by the Ethics Committee of the Bambino Gesù Children’s Hospital according to the Declaration of Helsinki (as revised in Seoul, Korea, October 2008).

### Statistical analysis

Statistical analysis was performed using the software IBM SPSS (Statistical Package for the Social Sciences) version 24.0. The quantitative variables were described as mean and standard deviation (SD) (normally distributed) or median and interquartile range (not-normally distributed). Categorical variables were described as absolute and relative frequencies. Means were compared with the Student’s t-test, and in case of not-normally distributed the Mann Whitney test was used. The Chi-Square test was used to assess the association between categorical variables. Multivariable logistic regression analysis was used for estimation of risk factors associated with the BMI groups and to explore the association between the characteristics of children with injury diagnosis and BMI. The selection of independent variables was done by backward elimination starting with all predictors in the model and remaining the last significant variable for each step. The *p* value < 0.05 was considered statistically significant.

## Results

### Characteristics associated to BMI in our sample

From January 1th to October 31th 2013, 60,030 admissions were recorded at our pediatric ED, and of those, 13,267 were children aged 6–18 years old; a total of 265 was excluded because severally ill. Of the remaining sample, 2848 patients were consented to participating study by the parents, but 160 were excluded for having BMI < 5th.

The prevalence of normal weight, overweight and obesity in our cohort was 66.5%, 20%, 13.5%, respectively.

The characteristics of overall population related to BMI are listed in Table 1.

**Table 1:** Characteristics of children admitted to emergency department and bivariate associations between covariates and body mass index

Variable	Total *n* = 2688	BMI < 85th *n* = 1787 (66.5%)	BMI ≥ 85th - < 95th *n* = 538 (20%)	BMI ≥ 95th *n* = 363 (13.5%)	*P* value
Age group					
School children	1637 (60.9%)	1025 (57.4%)	352 (65.4%)	260 (71.6%)	< 0.001
Adolescents	1051 (39.1%)	762 (42.6%)	186 (34.6%)	103 (28.4%)	
Gender					
Male	1509(56.1%)	937 (52.4%)	312 (58%)	260 (71.6%)	< 0.001
Female	1179 (43.9%)	850 (47.6%)	226 (42%)	103 (28.4%)	
Ethnic groups					
Italian	2464 (91.7%)	1640 (91.8%)	491 (91.3%)	333 (91.7%)	0.93
Others	224 (8.3%)	147 (8.2%)	47 (8.7%)	30 (8.3%)	
Symptom onset (days)					
(mean ± SD)	3.81 ± 7.1	3. 81 ± 7.2	3.82 ± 7.3	3.81 ± 6.3	0.09
Emergency triage code					
Yellow	141 (5.2)	96 (5.4%)	25 (4.6%)	20 (5.5%)	0.78
Green-White	2547 (94.8%)	1691 (94.6%)	513 (95.4%)	343 (94.5%)	
Number of emergency department visits per year					
(mean ± SD)	3.42 ± 3.74	3.35 ± 3.475	3.51 ± .311	3.65 ± 4.08	0.77
Abdomen circumference (cm)					
(mean ± SD)	73.27 ± 12.01	69.11 ± 9.26	77.90 ± 10.81	86.88 ± 13.11	< 0.001
Diagnostic categories					
Injury	746 (27.7%)	500 (27.9%)	154 (28.6%)	92 (25.3%)	0.79
All others	1942 (72.3%)	1282 (72.1%)	384 (71.4%)	271 (74.6%)	
Outcome					
Discharge	2458 (91.4%)	1637 (91.6%)	488 (90.7%)	333 (91.7%)	0.78
Hospitalization	230 (8.6%)	150 (8.4%)	50 (9.3%)	30 (8.3%)	
Length of hospital staying (days)					
(mean ± SD)	0.53 ± 5.4	0.37 ± 1.655	1.10 ± 11.506	0.41 ± 2.08	0.021

*BMI* Body Mass Index, *SD* standard deviation

Males were more frequently obese than other BMI groups (*p* < 0.001) and the school age children were more commonly obese than adolescents compared to other BMI groups (*p* < 0.001).

**Fig. 1:**
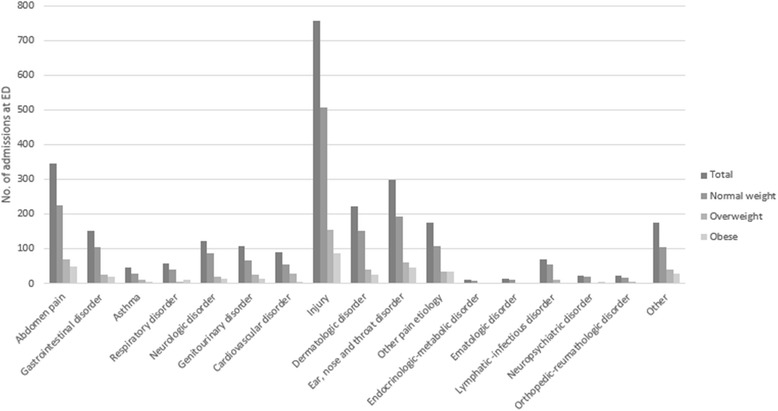
Distribution of various causes of admission to emergency department related to Body Mass Index groups

The causes of admissions to ED are illustrated in Fig. 1.

The multivariable logistic regression analysis (Table 2) showed that school age children were over 23 times significantly more likely to be obese (OR: 23.22; CI: 95%: 15.60–34.56; *p* < 0.001) and over 5 times to be overweight (OR: 5.17; CI: 95%: 3.96–6.74; *p* < 0.001) than adolescents compared to children with normal weight.

**Table 2:** Logistic regression analysis for predictive factors of obesity and overweight in children admitted to emergency department

Variable	Obese (BMI ≥ 95th) vs Normal weight ^a^			Overweight (≥ 85th- < 95th) vs Normal weight ^a^		
	β	OR	95% CI	β	OR	95% CI
Age group						
School children	3.14***	23.22	15.60–34.56	1.64***	5.17	3.96–6.74
Adolescents	–	1		–	1	
Gender						
Male	0.94***	2.56	1.86–3.51	0.26***	1.30	1.05–1.61
Female	–	1		–	1	
Abdomen circumference (cm)	0.22***	1.25	1.23–1.27	0.13***	1.13	1.12–1.15
Number of emergency department visits per year	0.06**	1.06	1.02–1.11	0.04*	1.04	1.01–1.07
Diagnostic category						
Injury	−0.41*	0.69	0.49–0.96	−0.14	0.87	0.68–1.10
Other diagnosis	–	1		–	1	
Length of hospital staying (days)	0.03	1.03	0.97–1.09	0.04	1.04	0.99–1.09

*OR* Odd ratio, *BMI* Body Mass Index, *CI* Confidence interval

*p < 0.05

**p < 0.01

***p < 0.001

^a^The reference category in the multinomial logistic model is those with normal weight (BMI 5th–85th)

Males were significantly over 2-fold at risk to be obese (OR: 2.56; CI: 95%: 1.86–3.51; *p* < 0.001) and 30% to be overweight (OR: 1.30; CI: 95%: 1.05–1.61; *p* < 0.001) respectively than females.

The children with obesity and overweight were more likely to report a major number of visits for year at ED (OR: 1.06; CI: 95%: 1.02–1.11; *p* < 0.001 and OR: 1.04; CI: 95%: 1.01–1.07; <= 0.05).

The children with obesity were 34% less likely to present injury compared to patients with normal weight (OR: 0.66; CI: 95%: 0.47–0.93; *p* < 0.05).

### Association between BMI and injury in children admitted to ED

In our population, 746 (27.7%) children admitted to ED had a diagnosis of injury. The characteristics of injury subset are reported in Table 3.

**Table 3:** Characteristics of injury subset at emergency department and bivariate associations between covariates and body mass index

Variable	Total *n* = 746	BMI < 85th *n* = 500 (67%)	BMI ≥85th–95th *n* = 154 (20.6%)	BMI ≥95th *n* = 92 (12.3%)	*P* value
Age group					
School children	466 (62.5%)	293 (58.6%)	106 (68.8%)	67 (72.8%)	0.007
Adolescent	280 (37.5%)	297 (41.4%)	48 (31.2%)	25 (27.2%)	
Gender					
Male	452(60.6%)	288 (57.6%)	95 (61.7%)	69 (75%)	0.007
Female	294(39.4%)	212 (42.4%)	59 (38.3%)	23 (25%)	
Emergency triage code					
Yellow	685 (91.8%)	469 (93.8%)	145 (94.2%)	84 (91.4%)	0.61
Green-White	61 (8.2%)	31 (6.2%)	9 (5.8%)	8 (8.7%)	
Abdominal Circumference (cm)					
(mean ± SD)	73.8 8 ± 12,13	70.05 ± 9.65	78.64 ± 10.97	86.73 ± 14.38	< 0.001
Outcome					
Discharge	685 (91.8%)	468 (93.6%)	140 (90.9%)	77 (83.7%)	0.006
Hospitalization	61 (8.2%)	32 (6.4%)	14 (9.1%)	15 (16.3%)	
Length of hospital stay (days)					
(mean ± SD)	0.31 ± 1.4	0.27 ± 1.21	0.3 ± 1.08	0.6 ± 1.56	0.78
Injured body region					
Head/neck	46 (6.2%)	33 (6.6%)	9 (5.8%)	4 (4.3%)	0.08
Face	55 (7.4%)	38 (7.6%)	15 (9.7%)	2 (2.2%)	
Upper extremity	385 (51.6%)	257 (51.4%)	79 (51.3%)	49 (53.3%)	
Lower extremity	206 (27.6%)	135 (27.0%)	42 (27.3%)	29 (31.5%)	
Trunk	48 (6.4%)	33 (6.6%)	8 (5.2%)	7 (7.6%)	
Multiple site	6 (0.8%)	4 (0.8%)	1 (0.6%)	1 (1.1%)	
Types of injuries					
Fracture	260 (34.9%)	173 (34.6%)	48 (31.2%)	39 (42.4%)	0.13
Contusion/abrasion	282 (37.7%)	195 (39.0%)	62 (40.3%)	24 (26.1%)	
Sprain/strain/dislocation	108 (14.5%)	66 (13.2%)	25 (16.2%)	17 (18.5%)	
Laceration	42 (5.6%)	28 (5.6%)	8 (5.2%)	6 (6.5%)	
Concussion	31 (4.2%)	23 (4.6%)	6 (3.9%)	2 (2.2%)	
Multiple trauma/other mechanism	24 (3.2%)	15 (3%)	5 (3.2%)	4 (4.3%)	
Injury mechanism					
Fall/crash at leisure-sport activities	494 (66.2%)	335 (67%)	100 (64.9%)	59(64.1%)	0.023
Road traffic injury	16 (2.1%)	9(1.8%)	4 (2.6%)	3 (3.3%)	
Fall between planes	27 (3.6%)	22 (4.4%)	4 (2.6%)	1(1.1%)	
Static and passive mechanism	31 (4.2%)	31 (4.2%)	3 (1.9%)	10(10.9%)	
Unknown mechanism	78 (23.9%)	116 (23.2%)	43 (27.9%)	19(20.7%)	
Fracture Location					
Lower extremity	44 (16.9%)	27(15.6%)	7 (14.6%	10 (25.6%)	0.21
Upper extremity	199(76.5%)	134 (77.5%)	37 (77.1%)	28 (71.8%)	
Other	17 (6.5%)	12 (6.9%)	4 (8.3%)	1 (2.6%)	
Dislocated Fracture	76 (10.2%)	44 (8,8%)	15 (9.8%)	17 (18.5%)	0.019

*BMI* Body Mass Index, *SD* standard deviation

The prevalence of the injury was 67%, 20.6%, 12.3%, in children with normal weight, overweight and obesity respectively. The obese injured patients were more frequently school age (*p* = 0.007) and males (*p* = 0.007). Obese children were hospitalized more than other BMI groups (*p* = 0.006).

The fracture occurred mostly in obese (42.4%) but not significantly. The dislocated fracture was significantly common in patients with obesity (18.5%) than overweight (9.8%) and normal weight (8.8%) (*p* = 0.05). Concerning the injury mechanism, the fall/crash at leisure-sport activities occurred more frequently (66.2%) and pertained to normal weight children compared to others (*p* = 0.023).

The multivariate analysis exploring the association between the clinical characteristics of the injury subset and the BMI groups is showed in Table 4. The likelihood of being obese increased by over 22 times in school age than adolescents (OR: 22.94; CI: 10.50–50.14; *p* < 0.001); similarly, school age children were over 6 fold at risk to be overweight than adolescents (OR: 6.45; CI: 95%: 3.71–10.97; *p* < 0.001). Males had a higher risk to be obese (OR: 2.43; CI: 95%: 1.26–4.67; *p* < 0.01). Considering the outcome, obese children were less likely to be discharged (OR: 1.13: CI: 0.12–0.73; *p* < 0.01). The static and passive mechanism was over 3 fold more likely to occur in the group of the obesity (OR: 3.36; CI: 1.35–8.38; *p* < 0.01).

**Table 4:** Logistic regression analysis for predictive factors of obesity and overweight in injured patients

Variable	Obese (BMI ≥95th) vs Normal weight^a^			Overweight (≥85th- < 95th) vs Normal weight^a^		
	β	OR	95% CI	β	OR	95% CI
Age group						
School age	3.13***	22.94	10.50 –	1.87***	6.45	3.71–10.97
Adolescents	–	1	50.14	–	1	
Gender						
Male	0.89**	2.43	1.26–4.67	0.29	1.34	0,88–2,05
Female	–	1		–	1	
Abdomen circumference (cm)	0.21***	1.23	1.20–1.27	0.120***	1.13	1.10–1.16
Injury mechanism						
Fall/crash at leisure-sport activities	0.64	1.07	0.61–1.86	−0.22	0.80	0.53–1.21
Road traffic injury	0.70	2.02	0.50–8.13	0.17	1.20	0.35–4.06
Fall between planes	−1.30	0.27	0.03–2.16	−0.72	0.49	0.16–1.49
Static and passive mechanism	1.12**	3.36	1.35–8.38	−1.21	0.30	0.07–1.33
Unknown mechanism	–	1		–	1	
Outcome						
Discharge	−1.23**	0.29	0.12–0.73	−0.43	0.65	0.31–1.37
Hospitalization	–	1		–	1	
Injury and age group						
Fracture in school age	0.90 **	2.46	1.28–4.74	0.271	1.32	0.75 2.37
Other injuries in school age		1.81	0.97–3.38	0.67**	1.95	1.21–3.15
Fracture in adolescents	0.59	1.26	0.53–2.99	0.29	1.34	0.70–2.60
Other injuries in adolescents	0.23-	1		–	1	
Injury and body region						
Fracture at upper extremity	0.43	1.54	0.76–3.13	0.43	0.91	0.53–1.59
Other injuries at upper extremity	0.23	1.26	0.69–2.65	0.23	1.13	0.66–1.95
Fracture at lower extremity	1.01*	2.73	1.08–6.92	1.01*	0.86	0.34–2.17
Other injuries at lower extremity	0.26	1.30	0.61–2.80	0.26	1.07	0.62–1.88
Fracture at other body regions	−4,86	0.61	0.07–5.13	−4,86	1.10	0.33–3.68
Other injuries at other body regions	–	1		–	1	
Type fracture						
Dislocated fracture	1.14*	3.13	1.16–8.48	0.47	1.59	0.74–3.43
Compound fracture	–	1		–	1	

*OR* Odd ratio, *BMI* Body Mass Index,*CI* Confidence interval

*p < 0.05

**p < 0.01

***p < 0.001

^a^The reference category in the multinomial logistic model is those with normal weight (BMI 5th–85th)

The fracture in school age group was over 2-fold more likely to involve obese children (OR: 2.46; CI: 1.28–4.78; *p* < 0.01). The odds of reporting a fracture at lower extremity for children with obesity were over twice than normal weight (OR: 2.73; CI: 1.08–6.92; *p* < 0.05); similarly, children with overweight were 20% more at risk to report a fracture at lower extremity (OR: 0.86; CI: 0.34–2.17; *p* < 0.05).

The dislocated fracture was over 3-fold more likely to occur in obese and overweight (OR: 3.13; CI: 1.16–8.48; *p* < 0.01).

## Discussion

In our study, the prevalence of overweight and obesity was reported to be 20.2% and 13.5% respectively. The regional (Lazio) data from national surveillance obesity system shows a prevalence of overweight and obesity respectively 21.7% and 9.4% in 2014 [9]. Our data derived from the ED admissions of a tertiary hospital, although not representative of the Lazio region, and therefore not comparable, show high levels still worrying of excess of weight body in children.

Concerning epidemiological aspects, school age children were significantly more likely to be obese or overweight than adolescents. The early childhood is a key window period for delivering potentially effective preventive interventions because these years are a period of sensitivity to environmental influences and for the imprinting of health lifestyle [10]. Moreover, the excess of body weight in younger children is a highly predictive of obesity during adolescence and adulthood [11, 12]. Males were at a higher risk to be obese and overweight than females. This finding is in line with national and regional data [9]. No difference was showed between Italian and children with different ethnic background in contrast with a previous study conducted in Emilia Romagna Region in which the obesity resulted to be higher among Immigrants [13]. Probably in our region children from immigrant families abandon increasingly the originating eating habits to assimilate those of the host country [14]. The obese and overweight children were more likely to report a major number of visits for year at ED than patients with normal weight. Some studies have reported that excess body weight disorders involve an increased health care utilization, including ED visits^1^ for both acute and routine medical care [4]. This phenomenon might be due to increase of obesity related comorbidities and consequently implicates a growing burden on healthcare system [15]. Nevertheless, this finding might to be due to confounding factors such as the family and social class that we did not considered in our analysis. Although in different settings from the one where we carried out our research and with weak association measures, some researcher reported that adjusting for demographics, socio-economic status, and chronic medical conditions, overweight or obese children still more frequently utilize the ED and outpatient clinic visits than under/healthy weight children [16]. Regarding causes of admission to ED, obese children were 34% less likely to present an injury. The possible relationship between obesity and injury is a relatively new interesting area of study, although the research in this area is limited for children. An association between obesity and various types of injury is described such as head and chest injuries in motor vehicle crashes [17], musculoskeletal injuries [18], ankle injuries [19], lower extremities [8]. Probably in our study children with BMI ≥ 85th were less likely to report an injury because of the type of involved injury mechanism. In fact, the fall/crash leisure-sport activities occurred more frequently in normal weight whereas the passive and static mechanism occurred mostly in BMI group ≥95th. The static and passive mechanism occurred mostly in obese children. These findings might indicate that normal weight children were exposed to a higher injury risk because of a major physical activity.

Among different types of injury, the fracture in school age individuals was over twice more likely if obese. There are few potential explanations for this data. First, age-related variation in mobility patterns should be considered. In fact, physical activity decreases with advancing age during childhood [20], because older children spend more time in sedentary behaviors including screen based (i.e. T*V*/*v*ideo viewing, playing PC/video games etc.) and not screen based (i.e. texting, talking on the phone, etc.) [21], in particular overall sedentary time and individual sedentary behaviors increase during the transition from primary/middle to secondary/high school [22]. Finally, adolescents with obesity are supposed to present an increased bone density, similar to the pattern seen in obese adults [23].

Among different types of injury, the fracture occurred mostly in obese children but not significantly.

Children with obesity and overweight were more likely at risk for fracture at lower extremity (*p* < 0.05). This finding is in line with other studies that show the association between childhood obesity and increased risk of lower extremity fracture [23]. We might explain this finding by interaction of different potential factors. Firstly, the mechanical effect involves the overloading of bones, joints and soft tissues of the locomotor system on lower extremity influencing the alignment, structure, function of the hip, knee and foot [24], and consequently making more vulnerable the bone and joint of lower extremity to a potential risk of fracture and osteoarthritis [25]. Secondly, biomechanical factors might be considered, such as effects of excess body weight on the walking gait. In fact, children with excess weight disorders report a slower habitual or self-selected walking speeds with a concomitant reduction in step length and step frequency [26]. Thus, the reduced walking speed is typified by longer single support and double support phase durations along with a shorter swing phase [27]. The consequence of excess mass in children is an increase in both the absolute amount of force applied to the joint and the muscular force needed to move the additional mass during ambulation [27]. All these mechanical and biomechanical factors potentiate the effect of obesity on decreased bone density [28]. The bone density not only is affected by reduced physical activity or by bone development that is overtaxed by rapidly increasing weight [28], but we suppose that the vitamin D deficiency might play a decisive role in the pathogenesis of lower extremity fracture in children with BMI ≥ 85th. A previous cross-sectional study, carried out in our tertiary care center for obesity at Children’s Hospital Bambino Gesù, reports the vitamin D levels are inversely associated with nonalcoholic steatohepatitis and fibrosis in children with nonalcoholic fatty liver disease [29] that is a complication in children with obesity. Since this is the exclusive experience of a tertiary center, we might presume that children enrolled in this study might present characteristic similar to our cohort because both of them belong to the same hospital’s catchment area. This hypothesis might represent a powerful stimulus for a future cross sectional study in order to detect the potential rule of vitamin D in children with excess weight disorders presenting to our hospital with the lower extremity fracture.

A series of limitations should be considered in our study. First, as this study was carried out in a tertiary care center, its findings may not be extended to the general population. Second, the anthropometric parameters were measured by health providers instructed but we did not consider the error margin of measurement determined by the experience of the providers and by the compliance of the patient. Third, we did not consider chronic or underlying diseases and ongoing therapeutic treatments, thus we cannot exclude this potential confounder.

## Conclusion

Our results indicate that school age children presenting to ED are more at risk of obesity and overweight than adolescents. Although obese children are less at risk of injury, probably because less prone to leisure-sport activities than normal weight counterpart, fractures occur more frequently in school age children with obesity. The childhood is a crucial temporal window for implementation of effective prevention strategy and to avoid future complications. Therefore, the ED might be a strategic setting for in the initial screening of these children and parental counseling, and for a timely specialist referral of these children, especially if school age, in order to guarantee further weight control-related interventions.

## Abbreviations

BMI: Body Mass Index
CI: Confidence Interval
ED: Emergency Department
OR: Odd Ratio
SD: Standard Deviation
WC: Waist Circumference
